# Les moustiques (Diptera : Culicidae) de Djibouti : revue bibliographique (1970-2023)

**DOI:** 10.48327/mtsi.v4i1.2024.365

**Published:** 2024-03-12

**Authors:** Abdoulgabar ABDOURAHMAN OMAR, Oumnia HIMMI

**Affiliations:** Centre de recherche en géophysique, patrimoine naturel et chimie verte (GEOPAC); Laboratoire géo-biodiversité et patrimoine naturel (GEOBIOL) Institut scientifique, Université Mohammed V de Rabat, Maroc

**Keywords:** Culicidae, Inventaire, Maladies à transmission vectorielle, Moustiques, Revue bibliographique, Djibouti, Afrique subsaharienne, Culicidae, Inventory, Vector-borne diseases, Mosquitoes, Bibliographical review, Djibouti, Sub-Saharan Africa

## Abstract

**Introduction - Justification:**

L'histoire des moustiques à Djibouti à partir de la littérature scientifique a été retracée et synthétisée.

**Matériel et méthode:**

Une recherche exhaustive sur des bases de données bibliographiques électroniques (PubMed, Google Scholar, etc.) a été effectuée. Un filtrage des listes de références a été effectué pour accéder à des articles supplémentaires afin d'avoir plus des données.

**Résultats:**

À Djibouti, 37 espèces de moustique ont été répertoriées dont au moins 8 espèces sont potentiellement des vecteurs d'agents pathogènes responsables de maladies telles que le paludisme, la dengue, la fièvre jaune, le virus du Nil occidental et le chikungunya.

**Discussion - Conclusion:**

L'ambition était de pouvoir documenter l'apparition de nouvelles espèces de moustiques, la réapparition et la disparition d'autres espèces de moustiques, vecteurs et potentiels vecteurs d'agents infectieux importants pour la santé humaine ou animale sur le territoire. Les résultats obtenus vont permettre de documenter, guider et faciliter la consultation ultérieure de la base de données concernant la thématique traitée.

## Abstract (en)

**Background and justification:**

The Republic of Djibouti is located in the Horn of Africa, on the Gulf of Aden and the Bab-el-Mandeb detroit, at the southern entrance to the Red Sea. Prior to its independence in 1977, the Republic of Djibouti was known by two names: “Côte française des Somalis” until 1967, then “Territoire Français de Afars et Issas”. As part of our doctoral research on the ecology of mosquitoes in Djibouti, we noted a lack of information on the species encountered, and felt it essential to draw up a list of species before embarking on ecological monitoring. The aim of this work is to survey publications on mosquitoes in Djibouti and to synthesize data from this scientific literature in order to update the national inventory of Culicidae.

**Materials and methods:**

An exhaustive search of electronic bibliographic databases (PubMed, Scopus, HAL Open Archive, Science Direct and Google Scholar) was carried out. Reference lists were filtered to access additional articles in order to obtain more data. Two keywords were used: “Djibouti” and “French Territory of Afars and Issas”. A selection of scientific publications on Djibouti mosquitoes and/or diseases transmitted by mosquito vectors was made. Researches were conducted in articles selected. The names of the species listed were checked and validated by referring to the site Mosquito Taxonomic Inventory.

**Results:**

A total of 13 studies, published between 1970 and 2023, were found. Over the years, the composition of the Culicidae fauna has become well known. In part, the movement of people traveling to and from neighboring countries has been linked to the detection of new species and the reappearance of mosquito species in Djibouti. Numerous studies have been carried out over the years, including purely taxonomic studies and others focusing on the incrimination of mosquito vectors and the characterization of the pathogens they transmit. A total of 37 species, belonging to two subfamilies (Anophelinae and Culicinae), of mosquitoes divided between 7 genera *(Aedes, Anopheles, Culex, Culiseta, Lutzia, Mimomyia* and *Uranotaenia)* have been mentioned across the country. The number of species per genus is distributed as follows: 5 species of *Aedes* including 1 subspecies, 14 species of *Anopheles* including two subspecies, 12 species of *Culex* including 1 subspecies, 1 species for each of the genera *Culiseta* and *Lutzia* and finally 2 species respectively for the genera *Mimomiya* and *Uranotaenia.* Five species have been incriminated as vectors of diseases such as malaria, dengue fever, yellow fever, West Nile virus and chikungunya. Others are known for their potential role in pathogen transmission, including Zika and Rift Valley virus.

**Discussion - Conclusion:**

The bibliographical research enabled us to summarize the research carried out over more than half a century in the history of Djibouti, and to update the inventory of the country's mosquitoes, which now includes 37 species. Species names were reviewed and updated, and the case of *Anopheles gambiae* was also addressed. Two species mentioned as part of the Culicidae fauna of Djibouti appeared to be doubtful and are up for discussion. These results provide a useful information base for defining vector control priorities in Djibouti. They will also inform, guide and facilitate future consultations of our database. In addition, this study will help to identify research ways on mosquitoes in Djibouti.

## Introduction

À l'instar des autres pays d'Afrique, les moustiques sont connus à Djibouti pour être responsables de la transmission d'un certain nombre de maladies vectorielles, notamment le paludisme et la dengue. Dans le cadre de recherches doctorales sur l’écologie des moustiques dans ce pays, nous avons constaté un manque de capitalisation concernant les espèces rencontrées. Dresser en premier lieu un inventaire nous a paru primordial pour lancer un suivi écologique. Ce travail a pour but de recenser les publications qui se sont intéressées aux moustiques à Djibouti et de synthétiser les données de cette littérature scientifique pour dresser une liste nationale actualisée des Culicidae.

## Présentation de la région de l’étude

**Figure 1:**
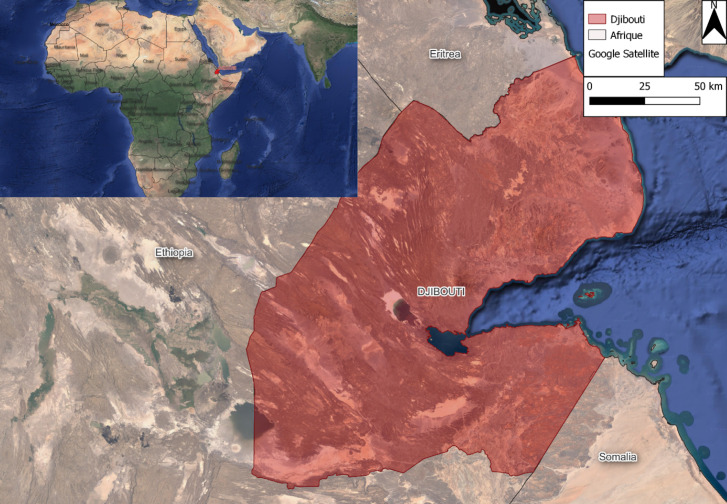
Carte de la République de Djibouti Map of the Republic of Djibouti

La République de Djibouti est située dans la Corne de l'Afrique face au détroit de Bab-el-Mandeb, à l'entrée de la mer Rouge. Avant son indépendance, en 1977, elle a eu deux appellations : Côte française des Somalis jusqu'en 1967, puis Territoire français des Afars et Issas (TFAI). Elle est limitrophe de la Somalie au sud-est, de l’Éthiopie au sud et à l'ouest, de l’Érythrée au nord et possède une frontière maritime avec le Yémen (Fig. 1). Djibouti présente un climat tropical aride caractérisé par l'irrégularité et la faiblesse des précipitations avec une moyenne de 95 mm par an, des températures élevées durant toute l'année qui réduisent le nombre de cours d'eau pérennes et provoquent une évaporation intense [19].

## Méthodologie

Cinq bases de données bibliographiques électroniques ont été consultées pour ce travail : PubMed, Scopus, Archive Ouverte HAL, ScienceDirect et Google Scholar. Deux mots clés géographiques ont été utilisés : « Djibouti », « French Territory of Afars and Issas ». Une sélection des publications scientifiques portant sur les moustiques de Djibouti et/ou les maladies vectorielles transmises par ces derniers a été effectuée.

Des articles mentionnés dans ces derniers ont été cherchés suite à un travail de filtrage des listes de références. Les noms des espèces répertoriées ont été vérifiés et validés en se référant sur le site suivant : Mosquito Taxonomic Inventory (MTI) (taxonomic-inventory.myspecies.info).

## Revue bibliographique

Dans les années 1950, 2 études citent 2 espèces de moustiques, respectivement *Anopheles arabiensis* [22] en provenance de Dire-Daoua vers Djibouti et une simple présence d'Aedes *albopictus* [31] dans le pays. Depuis cette période et avant les années 1970-1971, aucun entomologiste médical n'avait travaillé dans le territoire [16].

Des travaux sur l'entomologie médicale ont commencé à partir de 1970 par une vaste enquête sur les populations de moustiques de Djibouti effectuée par 2 entomologistes [7] suivie par une recherche sur *Aedes aegypti* en 1971 en Somalie et à Djibouti. Une enquête entomologique sur les insectes d'intérêt médical, potentiels vecteurs d'arbovirus, a été publiée en 1976 [23] suivie d'un premier inventaire des espèces du genre *Culex* trouvées entre 1973 et 1974 [5]. L’étude de F. Rodhain en 1976 a permis l'identification de 25 espèces de moustiques. Les données de ce travail ont initié un débat autour des modalités épidémiologiques de certaines arboviroses, constituant une étape importante dans la connaissance de la faune culicidienne de Djibouti. Elles ont permis la confirmation de l'identification d'une vingtaine d'espèces réparties entre 6 genres culicidiens *(Anopheles* [24], *Culex* [25] et *Aedes, Culiseta, Mimomyia, Uranotaenia* [26]). Carteron *et al.,* ont démontré une correspondance significative entre la présence *dAn. gambiaes l* et les cas autochtones du paludisme observés dans le territoire [4] ainsi que l'intérêt des insecticides imagocides [5, 12].

Dans les années 1990, Mouchet *et al.,* mentionnaient que la faune anophélienne est composée essentiellement d'An. *dthali,* espèce considérée non vectrice tout en indiquant la pénétration fréquente d'An. *arabiensis* sans y faire souche dans le territoire [17]. Rodier *et al,* affirmaient la distribution et l'abondance dAe. *aegypti* dans plusieurs quartiers de Djibouti-Ville [27]. Des travaux ont mentionné la pullulation d'An. *arabiensis* dans la région d'Ambouli dans toutes les collections d'eau [3, 18]. Faulde & Ahmed ont détecté pour la première fois *Culex pipiens* ssp. *torridus* avec la détection d'ARN viral du West Nile. *An. sergentii* a été également retrouvé, mais ces femelles se sont avéré être négatives pour *Plasmodium falciparum* et *Plasmodium vivax* [8]. En 2012, la circulation du virus West Nile a été observée chez *Cx. quinquefasciatus* et *Cx. pipiens* ssp. *torridus,* le premier assurant la transmission dans le milieu périurbain et rural et le second au niveau de l'environnement urbain [10].

Dans le rapport OMS sur le paludisme en 2013, *An. gambiae* et *An. arabiensis* sont mentionnés comme deux vecteurs principaux du paludisme à Djibouti [21]. Divers travaux de l'Institut National d'Hygiène de Santé Publique ont contribué à l’évaluation de la résistance des anophèles [15]. En 2014, *An. stephensi* a été trouvé à Djibouti et en Afrique avec son possible rôle dans une épidémie anormale du paludisme [9]. Seyfarth *et al.,* ont affirmé sa détection durant toute l'année et sa forte adaptation à son nouvel environnement [29]. De Santi *et al.,* ont mis en évidence le partage d'un même site de reproduction entre *An. stephensi, Ae. aegypti* et *Cx. quinquefasciatus* et montré qu'An. *stephensi* pourrait être responsable d'une augmentation du paludisme dans le pays [28].

Une surveillance entomologique de janvier à avril 2017 a permis la collecte des espèces *An. gambiae*; *An. dthali*; *An. stephensi*; *An. azaniae*; *An. nili somalicus*; *Ae. aegypti*; *Cx. quinquefasciatus*; *Cx. decens*; *Cx. nebulosus*; *Cx. pipiens; Cx. fatigans; Ur. bilineata* [11]. Une nouvelle espèce, *Aedes vexans* présentant un intérêt médical a été signalée sur le territoire et décrite comme une nuisance importante [33].

## Discussion

L'inventaire des espèces de moustiques citées à Djibouti (Tableau I) est de 37 espèces réparties entre 7 genres (*Aedes, Anopheles, Culex*, *Culiseta*, *Lutzia*, *Mimomyia*, *Uranotaenia*). Le genre *Anopheles* présente un total de 14 espèces dont 2 sous-espèces : *An. dthali, An. arabiensis*, *An. azaniae*, *An. dancalicus* et *An. gambiae* [23]; *An. harperi*, *An. rhodesiensis*, *An. rhodesiensis* ssp. *rupicolus*, *An*. *salbaii*, *An*. *pharoensis*, *An. nili*, *An. sergentii*, *An*. s*ergentii* ssp. *macmahoni* et *An. turkhudi* [1] *; An. stephensi* [9] et *An. somalicus* [11].

**Tableau I:** Liste des moustiques de Djibouti List of mosquitoes in Djibouti

**Famille : Culicidae**
**Sous-famille : Anophelinae**
**Genre : *Anopheles***
**Sous-genre : *Cellia***
*Anopheles (Cellia) arabiensis* Patton, 1905 [22]
*Anopheles (Cellia) azaniae* Bailly-Choumara, 1960 [24]
*Anopheles (Cellia) dancalicus* Corradetti, 1939 [1]
*Anopheles (Cellia) dthali* Patton, 1905 [16)
*Anopheles (Cellia) gambiae* Giles, 1900 [23]
*Anopheles (Cellia) harperi* Evans, 1936 [1]
*Anopheles (Cellia) nili* (Theobald, 1904) [11]
*Anopheles (Cellia) pharoensis* Theobald, 1901 [24]
*Anopheles (Cellia) rhodesiensis* Theobald, 1901 [24]
*Anopheles (Cellia) rhodesiensis* ssp. *rupicolus* Lewis, 1937 [1]
*Anopheles (Cellia) salbaii* Maffi & Coluzzi, 1958 [24]
*Anopheles (Cellia) sergentii* (Theobald, 1907) [8]
*Anopheles (Cellia) sergentii* ssp. *macmahoni* Evans, 1936 [24]
*Anopheles (Cellia) somalicus* Rivola & Holstein 1957 [11]
*Anopheles (Cellia) stephensi* Liston, 1901 [9]
*Anopheles (Cellia) turkhudi* Liston, 1901 [16]
**Sous-famille : Culicinae**
**Tribu : Aedini**
**Genre : *Aedes***
**Sous-genre : *Stegomyia***
*Aedes (Stegomyia) aegypti* (Linnaeus, 1762) [23]
*Aedes (Stegomyia) albopictus* (Skuse, 1894) [31]
**Sous-genre : *Ochlerotatus***
*Aedes (Ochlerotatus) caspius* (Pallas, 1771) [23]
**Sous-genre : *Aedimorphus***
*Aedes (Aedimorphus) vexans* (Meigen, 1830) [33]
*Aedes (Aedimorphus) vexans* ssp. *arabiensis* (Patton) [23]
**Sous-genre : *Fredwardsius***
*Aedes (Fredwardsius) vittatus* Bigot, 1861 [23]
**Tribu : Culicini**
**Genre : *Culex***
**Sous-genre : *Oculeomyia***
*Culex (Oculeomyia) bitaeniorhynchus* Giles, 1901 [37]
**Sous-genre : *Culex***
*Culex (Culex) decens* Theobald, 1901 [25]
*Culex (Culex) laticinctus* Edwards, 1913 [25]
*Culex (Culex) pipiens* Linnaeus, 1758 [11]
*Culex (Culex) pipiens* ssp. *torridus* Iglisch, 1977 (8)
*Culex (Culex) quinquefasciatus* Say, 1823 [11]
*Culex (Culex) simpsoni* Theobald, 1905 [25]
*Culex (Culex) sitiens* Wiedemann, 1828 [16]
*Culex (Culex) tenagius* Cunningham van Someren, 1945 [25]
*Culex (Culex) thallasius* Theobald, 1903 [37]
*Culex (Culex) tritaeniorhynchus* Giles, 1901 [25]
*Culex (Culex) univittatus* Theobald, 1901 [25]
**Sous-genre : *Culiciomyia***
*Culex (Culiciomyia) nebulosus* Theobald, 1901 [11]
**Genre : *Lutzia***
**Sous-genre : *Metalutzia***
*Lutzia (Metalutzia) tigripes* de Grandpre & de Charmoy, 1900 [16]
**Tribu : Culisetini**
**Genre : *Culiseta***
**Sous-genre : *Allotheobaldia***
*Culiseta (Allotheobaldia) longiareolata* (Macquart, 1838) [26]
**Tribu : Ficalbiini**
**Genre : *Mimomyia***
**Sous-genre : *Etorleptiomyia***
*Mimomyia (Etorleptiomyia) mediolineata* (Theobald, 1904) [26]
**Sous-genre : *Mimomyia***
*Mimomyia (Mimomyia) mimomyiaformis* (Newstead, 1907) [26]
**Tribu : Uranotaeniini**
**Genre : *Uranotaenia***
**Sous-genre : *Uranotaenia***
*Uranotaenia (Uranotaenia) balfouri* Theobald, 1904 [26)
*Uranotaenia (Uranotaenia) bilineata* Theobald, 1909 [11]

La faune restante des culicidés rassemble un total de 23 espèces, dont deux identifiées au niveau de la sous-espèce, réparties entre 6 genres : *Ae. vexans*, *Ae. vexans* ssp. *arabiensis*, *Ae. vittatus, Ae. caspius* et *Ae. aegypti*, *Cx. tritaeniorhynchus* et *Cx. univittatus* [23]; *Ae. albopictus* [31]; *Cx. pipiens* [11]; *Cx. pipiens* ssp. *torridus* [8]; *Cx. simpsoni*, *Cx. laticinctus*, *Cx. tenagius* et *Cx. decens* [25]; *Lt. tigripes* et *Cx. sitiens* [16]; *Cx. bitaeniorhynchus* et *Cx. thallasius* [37]; *Cs. longiareolata*, *Ur. balfouri, Mi. mimomyiaformis* et *Mi. mediolineata* [26]; *Ur. bilineata, Cx. quinquefasciatus* et

*Cx. nebulosus* [11]. Selon le ministère de la Santé de Djibouti, *An. arabiensis* diminue dans les gîtes larvaires au détriment d'An. *stephensi* qui semble dominer la faune anophélienne. L’évolution climatique pourrait participer à cette situation. L'espèce *An. nili*, dans un rapport du ministère de la Santé en 2020 [15], a été classée provisoirement comme un vecteur principal du paludisme. Hamon & Mouchet considéraient *An. nili* comme un vecteur secondaire du paludisme au sud du Sahara, mais ayant une importance régionale majeure avec la capacité d'assurer à lui seul la transmission du paludisme au moins dans certaines zones [13]. Un travail de révision et de confirmation serait nécessaire sur la sous-espèce *An. nili somalicus,* citée dans le travail sur la résistance des vecteurs du paludisme dans la région de la Méditerranée Orientale de l'OMS [1] comme faisant partie de la faune anophélienne de Djibouti. En effet, la base de données MTI montre que le complexe *An. nili* représente plusieurs espèces et entre autres *An. somalicus* qui a été cité comme étant rare et distribué dans les régions situées au nord de la République de Djibouti (régions de Tadjourah et Obock) [11]. Cependant est-elle la seule espèce qui représente le complexe *nili* à Djibouti ?

Depuis 2013, au sein du complexe d'espèces *An. gambiae,* deux nouvelles entités taxonomiques ont été reconnues et décrites sur la base de preuves moléculaires et bionomiques : *An. coluzzii* Coetzee & Wilkerson sp. n. (forme M) et *An. gambiae* Giles (forme S) [6]. Des travaux d'identification moléculaires à partir d'espèces considérées *An. gambiae* à Djibouti seraient intéressants.

En 1975 White mentionnait l'existence de la sous-espèce *Aedes vexans* ssp. *arabiensis* qui serait répartie dans la région éthiopienne notamment la Somalie [36]. En 1976, Rodhain mentionnait la présence d'Ae. *arabiensis* [23]. *Ae. arabiensis* ne serait pas une espèce mais plutôt une sous-espèce d'Ae. *vexans* ssp. *arabiensis. Ae. vexans* a été détecté par l'unité de recherche médicale de la Marine (NAMRU-3) sur le Camp Lemonnier (CLDJ) en octobre 2019 [33]. C'est une espèce connue comme un vecteur compétent pour le virus de la fièvre de la Vallée du Rift en Europe [2] et pour le virus du Nil Occidental [34]. *Ae. vexans* a également été trouvé à l'aérodrome de Chabelley Djibouti [CADJ] ainsi que dans un village au sud de la région d'Arta à Djibouti à moins de 13 km de la ville, suggérant qu'il est établi à Djibouti [33].

*Cx. sitiens,* identifié par Mouchet en 1971 [16] et par Rodhain *et al.* en 1977 [25] a été observé en décembre 2019. L'espèce est surtout connue par sa nuisance nocturne mais pas comme vecteur de maladie à Djibouti. Cependant, elle est susceptible d’être infectée après un repas sanguin sur un porteur de virus [14, 35]. *Culex fatigans* [ou *Culex pipiens* ssp. *fatigans*] a été placé en synonymie avec l'espèce *Culex quinquefasciatus* par Stone [30]. L'espèce *Cx. tigripes* [16] citée par Mouchet J, en 1971, a été classée depuis 2003 par Tanaka dans le genre *Lutzia* et est aujourd'hui nommée *Lutzia tigripes* [32]. Des espèces signalées dans la littérature peuvent être considérées douteuses. Une espèce dont l'identification signalée par Rodhain [25], *Cx. ethiopicus* Edwards 1912 est le synonyme de *Cx. bitaeniorhynchus* Giles, 1901 citée récemment appartenant à la faune culicidienne de Djibouti [37]. Étant citée au minimum deux fois, dont une dernière mention très récemment, cette espèce est potentiellement présente sur le territoire de Djibouti. *Ae. albopictus* (Skuse, 1894) a été signalé une seule fois à Djibouti sans que l'origine de la citation ne soit claire [31]. Sa présence est probable vu son caractère envahissant et l'augmentation rapide de son aire de répartition mondiale notamment en Afrique centrale à partir de 2000 [20]. Toutefois aucune autre étude supplémentaire depuis cette première mention n'est venue confirmer sa présence à Djibouti.

## Conclusion

La synthèse bibliographique des travaux de recherche recensés durant plus d'un demisiècle d'histoire des moustiques de Djibouti, a permis d'actualiser l'inventaire des moustiques du pays. Ces résultats constitueront un socle d'informations utiles pour prioriser la lutte antivectorielle au regard des maladies que ces vecteurs peuvent transmettre. La carence relative en données entomologiques suggère que de nouveaux travaux de terrain soient entrepris dans l'intérêt de la santé humaine et animale.

## Contribution des auteurs

Abdoulgabar ABDOURAHMAN OMAR : prospection bibliographique, définition de la méthodologie et rédaction du manuscrit. Oumnia HIMMI : conception de l’étude, correction et validation du manuscrit.

## Remerciements

Nos remerciements sont adressés au Dr. Yahya Ali Ismael pour avoir traduit la partie Abstract en anglais.

## Conflit d'intérêt

Les auteurs ne déclarent aucun conflit d'intérêts.
